# Clinical characteristics and outcomes of continuous renal replacement therapy performed on younger children weighing up to 10 kg

**DOI:** 10.55730/1300-0144.5642

**Published:** 2023-02-14

**Authors:** Emrah GÜN, Anar GURBANOV, Özlem NAKİP SARITAŞ, Ahmet YÖNTEM, Ayşen DURAK, Edin BOTAN, Fevzi KAHVECİ, Serhan ÖZCAN, Ebru AZAPAĞASI, Serhat EMEKSİZ, Mutlu UYSAL YAZICI, Selman KESİCİ, Özden ÖZGÜR HOROZ, Ömer ERDEVE, Benan BAYRAKÇI, Dinçer YILDIZDAŞ, Tanıl KENDİRLİ

**Affiliations:** 1 Department of Pediatric Intensive Care, Faculty of Medicine, Ankara University, Ankara, Turkey; 2 Department of Pediatric Intensive Care, Faculty of Medicine, Hacettepe University, Ankara, Turkey; 3 Department of Pediatric Intensive Care, Faculty of Medicine, Çukurova University, Adana, Turkey; 4 Department of Pediatric, Faculty of Medicine, Ankara University, Ankara, Turkey; 5 Department of Pediatric Intensive Care, Faculty of Medicine, Ankara City Hospital, Ankara, Turkey; 6 Department of Pediatric Intensive Care, Faculty of Medicine, Dr. Sami Ulus Gynecology Obstetrics and Child Health and Diseases Training and Research Hospital, Ankara, Turkey; 7 Department of Neonatology, Faculty of Medicine, Ankara University, Ankara, Turkey

**Keywords:** Continuous renal replacement therapy, children, pediatric intensive care unit

## Abstract

**Background/aim:**

This study aimed to investigate the clinical features, modality, complications, and effecting factors on the survival of children weighing up to 10 kg who received continuous renal replacement therapy (CRRT).

**Materials and methods:**

This study was a retrospective observational study conducted in five pediatric intensive care units in tertiary hospitals in Turkey between January 2015 and December 2019.

**Results:**

One hundred and forty-one children who underwent CRRT were enrolled in the study. The median age was 6 (range, 2–12) months, and 74 (52.5%) were male. The median weight of the patients was 6 (range, 4–8.35) kg and 52 (36.9%) weighed less than 5 kg. The most common indication for CRRT was fluid overload in 75 (53.2%) patients, and sepsis together with multiorgan failure in 62 (44%). The overall mortality was 48.2%.

**Conclusion:**

Despite its complexity, CRRT in children weighing less than 10 kg is a beneficial, lifesaving extracorporeal treatment modality.

## 1. Introduction

Continuous renal replacement therapy (CRRT) has been the preferred treatment method for critically ill children with acute kidney injury (AKI) and fluid overload (FO) [1]. The indications for CRRT in pediatric intensive care units (PICUs) are varied [2]. Diuretic-resistant fluid overload, metabolic acidosis, poisoning, electrolyte abnormalities, and attacks of inborn errors of metabolic disease are the most common reasons for CRRT use in PICU settings [2, 3]. The first report on CRRT use in adults was reported by Kramer et al. [4] in 1977, and Leone et al. [5] documented a successful fluid-electrolyte and acid-base balance with CRRT in anuric children in 1986 [5].

Among the renal replacement therapy (RRT) modalities, peritoneal dialysis (PD) is the most used method in neonatal intensive care units (NICU) due to its applicability and availability [6]. However, CRRT is more effective in terms of the removal of free water and ammonia, and it is preferred over PD, which has limited benefits in younger children who have undergone abdominal surgery recently [6, 7]. However, CRRT use in younger children brings some risks due to higher extracorporeal blood volumes in the circuit than the patient’s blood volume and relatively higher blood flows [8]. The main concerns about CRRT in younger children are establishing vascular access, hemodynamic instability during CRRT, hypothermia, and hemodilution [9]. These problems are more frequently observed in younger children, and it causes challenges in all centers; therefore, CRRT use in younger children can only be performed in developed and dedicated PICUs [9]. The improved technology of pump-driven volumetric-controlled CRRT devices with small extracorporeal volumes has increased the use of CRRT in younger children [10]. The purposes of this study were to determine the clinical characteristics of the patients and risk factors, which were mortality and CRRT-related complications, and the effecting factors on the survival of children weighing up to 10 kg who received CRRT.

## 2. Materials and methods

This was a retrospective, multicenter, descriptive study. We included children who weighed up to 10 kg of body weight and underwent CRRT between January 2015 and March 2021 in five PICUs. The study was approved by the Institutional Review Board of the Local University Faculty of Medicine (approval number: I10-667-21).

CRRT therapies were performed using PrismaflexTM HF20 Gambro (USA) and PrismaflexTM M60 Gambro (USA) sets. Two-lumen 6.5-Fr, 7-Fr, and 8-Fr dialysis catheters were used for vascular access in the patients. In patients undergoing extracorporeal membrane oxygenation (ECMO), the dialysis circuit was connected through the connectors on the venous lines of the ECMO circuit, not through a venous catheter. The following demographic and clinical data were collected: diagnosis, body weight, presence of organ failure, Pediatric Risk of Mortality (PRISM III) scores, Pediatric Logistic Organ Dysfunction (PELOD) scores, type of mechanical support [mechanical ventilator, extracorporeal membrane oxygenation (ECMO), therapeutic plasma exchange (TPE)], catheter insertion site [right or left internal jugular vein (RJVI, LVJI), femoral vein, subclavian vein], CRRT modality (continuous venovenous hemofiltration (CVVH), continuous venovenous hemodialysis (CVVHD), continuous venovenous hemodiafiltration (CVVHDF), CRRT circuit type (HF20, M60), circuit priming solution (red packed cell-normal saline mixture, albumin or normal saline), type of anticoagulation (heparin, citrate or no anticoagulation), ultrafiltration, number of circuits used, complete blood count and biochemistry results pre-CRRT and on the first day, use of blood products during CRRT, complications, vasoactive inotropic score (VIS), duration of CRRT, and outcomes (survivors, nonsurvivors). After enrollment, patients were further analyzed in two groups according to their body weight: less than 5 kg, and between 5 kg and 10 kg.

Acute kidney injury was categorized according to the RIFLE (Risk, Injury, Failure, Loss, and End-stage Kidney) and pediatric RIFLE (pRIFLE) criteria according to serum creatinine, estimated creatinine clearance (eCCl), and urine output within 24 h before the initiation of CRRT [11]. Fluid overload was calculated using the following equation:

Fluid overload (%) = (Fluid in – fluid out)/admission weight in PICU × 100%. Multi-organ dysfunction syndrome (MODS) was defined as the ‘involvement of three or more organ systems’ [12].

The PRISM III scores were recorded, which were calculated at PICU admission. Indications for CRRT were categorized as follows: fluid overload (>10%), AKI, oliguria-anuria with severe sepsis, hyperkalemia (>7 mmol/L) with unresponsive to conventional treatments, metabolic acidosis unresponsive to conventional treatments, metabolic disease (hyperammonemia, methylmalonic acidemia), refractory hyper-hyponatremia, and electrolyte imbalance.

VIS was calculated using the following equation:

(VIS = dopamine dose [μg/kg/min] + dobutamine dose [μg/kg/min] + 100 × epinephrine dose [μg/kg/min] + 10 × milrinone dose [μg/kg/min] + 10,000 × vasopressin dose [U/kg/min] + 100 × norepinephrine dose [μg/kg/min]) [13].

Statistical analyses were performed using the SPSS v26.0 software package (Statistical Package for the Social Sciences for MacOS, SPSS Inc., USA). The patients were divided into survivors, nonsurvivors, and ‘<5 kg and 5–10 kg patients’. Numbers (n) and proportions (%) were used for descriptives of categorical variables. Means and standard deviations were used for normally distributed variables. The Mann-Whitney U test was used for comparison purposes as the continuous variables do not have a normal distribution with categorical variables having two categories. The results were reported as median values and interquartile ranges (IQR 25–75). Normal distribution was tested using histograms, the Shapiro-Wilk and Kolmogorov-Smirnov tests, and variation coefficients. The Wilcoxon test was used for related variables of the groups. The Chi-square test and Fisher’s exact test were used to compare nonnumerical parameters between categorical groups. The Kaplan-Meier method was used for survival analysis. P-values below 0.05 were accepted as statistically significant.

## 3. Results

We included 141 children with weights equal to or less than 10 kg who were treated with CRRT in this study. The patient distribution of the five centers was as follows: 37 (26.2%), 33 (23.4%), 32 (22.7%), 23 (16.3%), and 16 (11.3%) patients, respectively. The median age was 6 (range, 2–12) months, and 52.5% (n = 74) were male. The median weight of the patients was 6 (range, 4–8.35) kg and 36.9% (n = 52) weighed less than 5 kg. Twenty-one (14.9%) had primary renal disease, 36 (25.5%) had inborn error of metabolism disorders, 18 (12.8%) had cardiac disease, 35 (24.8%) had sepsis, 21 (14.9%) had pulmonary disease, four (2.8%) had liver disease, and six (4.3%) patients had other diseases. Among all the patients, the median PRISM III score was 17 (IQR 10–27). Respiratory failure (n = 92, 65.2%) was the most common organ insufficiency followed by renal (n = 83, 58.9%), cardiac (n = 80, 56.7%), hematologic (n = 45, 31.9%), neurologic (n = 38, 27%), and liver (n = 34, 24.1%). The most frequent indications for CRRT were FO in 75 (53.2%), and sepsis together with multiorgan failure in 62 (44%) patients. Other indications for CRRT and patients’ demographic data are given in Table 1.

The most common site for insertion of dialysis catheters was the RJVI in 87 (61.7%), followed by the femoral vein in 14 (9.9%), and the subclavian vein in four (2.8%), respectively. Sixteen (11.3%) patients were connected to ECMO circuits. The most common dialysis catheter size was 7 Fr (79.2%), followed by 6.5 Fr in 21 (16.8%), and 8 Fr in five (4%). Selected CRRT modalities were CVVHDF in 78 (55.3%) patients, CVVHD in 57 (40.4%), and CVVHF in six (4.3%) patients. These data are given in detail in Table 2.

The primary diagnosis, CRRT modality, and mortality rate of the children who weighed less than 5 kg and between 5 kg and 10 kg are shown in Table 3. When we compared the patients according to their weights, there were significant differences between those who weighed less than 5 kg and the 5–10 kg group regarding the presence of respiratory failure (80.7% vs. 56.1%, p = 0.03), renal failure (48% vs. 65.1%, p = 0.047), an inborn error of metabolism (51.9% vs. 49.4%, p = 0.003), ultrafiltration (67.3% vs. 79.7%, p = 0.036), hypotension (51.9% vs. 43.8%, p = 0.028), and invasive mechanical ventilation (94.2% vs. 74.1%, p = 0.003) (Table 3).

In laboratory parameters obtained before CRRT, a statistically significant difference was found between the survivors and nonsurvivors in respect of blood glucose (102 mg/dL vs. 134.5 mg/dL, p = 0.010), and uric acid levels (9.2 mg/dL vs. 6.1 mg/dL, p = 0.026), platelet count (182,000 10^3^/μL vs. 71,000 10^3^/μL, p < 0.001), serum lactate levels (3.7 mmol/L vs. 8.8 mmol/L, p < 0.001), and VIS (6.5 vs. 55, p < 0.001). On day 1 of CRRT, a statistically significant difference was found between the survivors and nonsurvivors in respect of potassium and hemoglobin levels, platelet counts, serum pH, bicarbonate and lactate levels, and VIS (Table 4).

Upon comparison of the laboratory parameters of patients before CRRT and on the first day of CRRT, a statistically significant difference was observed in the values of BUN, creatinine, potassium, uric acid, phosphorus, WBC, platelet, pH, HCO3, and ammonia (Table 5).

According to the characteristics of CRRT treatment, survivors and nonsurvivors showed significant differences in days of CRRT [1.5 (range, 0.85–3.35) vs. 2.5 (range, 1–5.25), p = 0.004)], type of circuit [HF20; 72 (98.6%) vs. 55 (80.8%), p = 0.001; M60; 1 (1.4%) vs. 13 (19.1%), p = 0.002)], ultrafiltration [48 (65.7%) vs. 58 (85.2%), p = 0.001], priming solutions [packed red cell (56.1% vs. 80.8%), p = 0.002], normal saline (42.4% vs. 13.2%, p < 0.001), albumin (1.4% vs. 5.8%, p = 0.148)], receiving platelets [26 (35.6%) vs. 55 (80.8%), p = 0.001] and FFP [28 (38.3%) vs. 50 (73.5%), p = 0.001)] during CRRT, and CRRT complications including hypotension (28.7% vs. 61.7%, p = 0.001) and hypothermia (28.7% vs. 44.1%, p = 0.002); these parameters are presented in Table 2.

One hundred and fifteen (81.6%) patients had invasive ventilation support, 23 (16.3%) patients underwent ECMO, and 42 (29.3%) patients were treated with TPE. The median length of PICU stay was 10 (IQR 4–25) days. The overall mortality was 48.2% (n = 68). The survivors and nonsurvivors showed significant age differences [8 (range, 4.25–14) months vs. 5 (range, 2–11) months), p = 0.026], weight [5 (range, 3.5–7.7) kg vs. 4 (range, 3.16–5.95) kg, p = 0.010], PRISM III scores [11 (range, 8–21.5) vs. 20 (range, 14.75–34.25), p < 0.001], PELOD scores [12 (range, 10–22.5) vs. 31 (range, 21–41.25), p < 0.001], and the number of organ system failures [1 (range, 1–3) vs. 4 (range, 3–4.75), p < 0.001].

**Figure 1:**
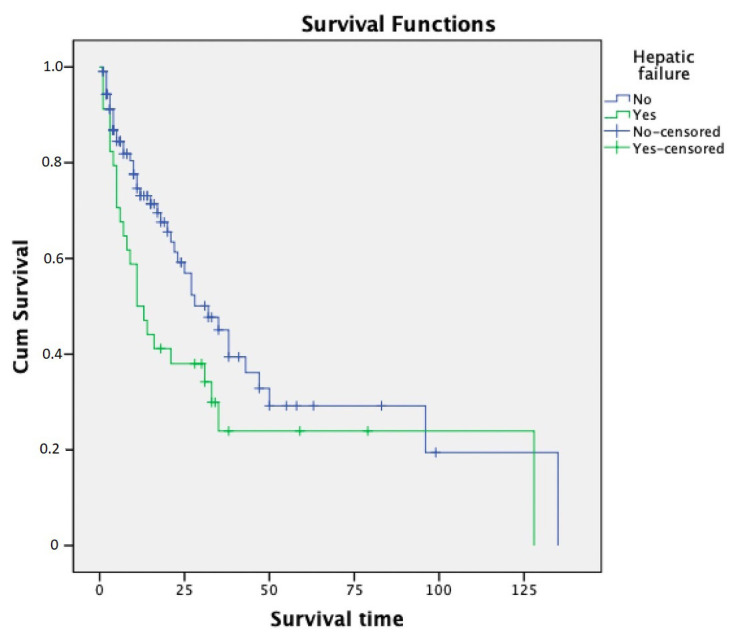
Relationship between survival time and hepatic failure (Kaplan-Meier test, p = 0.042).

**Figure 2:**
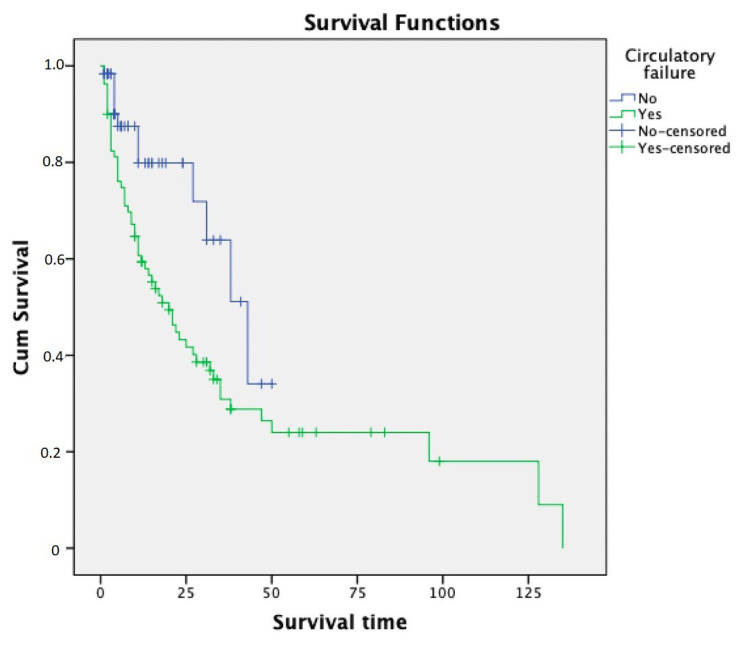
Relationship between survival time and circulatory failure (Kaplan-Meier test, p = 0.010).

Survival time analysis was calculated using the Kaplan-Meier method. The survival time showed significant differences in hepatic failure and circulation failure (p = 0.042 and p = 0.010, respectively); these data are presented in Figures 1 and 2. Survival times showed no significant differences in FO, body weight, sepsis, and ECMO support.

## 4. Discussion

We report our experience in 141 small children weighing less than or equal to 10 kg who were treated with CRRT. One of the most critical points required for CRRT in this extreme group is providing good vascular access for an efficient process. In a study on CRRT, especially in younger children, it was stated that the region used primarily for dialysis catheters was the left IJV under the guidance of ultrasonography [6, 9]. The neck veins are preferred sites for patients weighing less than 5 kg [14]. In the studies of Kaempfen et al. [13] and Symons et al. [10], the most frequently used catheter sites were the femoral vein in CRRT studies performed in children weighing less than or equal to 10 kg. Unlike their results, in this multicenter study, we observed that the RJVI was the most used site for vascular access. A comparison of catheter sites in patients weighing less than 5 kg and 5–10 kg showed that the most used catheter site was RJVI in both groups (59.6% and 62.9%, respectively), and there was no significant difference between the two groups.

The CRRT circuit onto the ECMO circuit becomes an advantage in children with low body weight, especially in patients without venous access [15, 16]. However, it has an increased risk of systemic inflammation and increased hemolysis due to flow turbulences [16]. We performed this technique on 16 patients who were on ECMO run without dialysis catheters.

Another critical element is the establishment of the CRRT circuit. High-volume sets are avoided because the blood volume is small in patients with low body weight [17, 18]. The blood volumes in devices such as CARPEDIEM (27 mL) and NIDUS (10 mL) used primarily for newborns are relatively low compared with other sets, but these CRRT devices are not available in most centers [18]. Conventional HD and CRRT devices are not approved for children weighing under 20 kg [19]. The NIDUS device is licensed for children weighing 0.8–8 kg [17,18]. CARPEDIEM can be used in children who weigh more than 2.5 kg and blood priming may not be required in children weighing up to 2.5 kg [18]. There are no CARPEDIEM and NIDUS devices in our centers. The most frequently used set (%) in our study was the Gambro Prismaflex HF20 set. The volume of this set is 60 mL, and it is recommended to prime with blood for babies weighing less than 5 kg [18]. In some patients, without the HF20 circuit, we had to use the M60 circuit. However, this rate was relatively low.

Circuit anticoagulation is an essential technical issue in CRRT, and its primary purpose is to prevent thrombotic processes when blood encounters the extracorporeal circuit [20]. Heparin is the most widely used anticoagulant [20]. Regional citrate anticoagulation (RCA) with citrate is another alternative anticoagulation method [3,20,21]. Compared with anticoagulation with heparin, RCA has been shown to reduce the risk of bleeding and improve circuit life [21–23]. Younger children who have undergone recent surgery often require RCA because of the postoperative condition or coagulopathy [22]. Despite increasing knowledge and experience in performing CRRT with RCA in the pediatric population, RCA in younger children remains a challenge because it increases the risk of citrate accumulation (mainly because of an imbalance between blood flow and body weight) [22]. To our knowledge, only a few studies have reported detailed data on RCA performance during CRRT in younger children, and a recent review suggested more reports were required on the use of citrate [22]. CARPEDIEM and NIDUS devices, which are specifically designed for younger children, do not yet provide automated RCA [18,24]. The standard anticoagulation method in our study was heparin. Although we prefer citrate in older children, we found that this rate is low for this age group.

CRRT has become a preferred treatment modality for AKI and fluid overload in critically ill pediatric patients in the last two decades [25]. CRRT has advantages over other renal replacement therapies (PD and IHD) [25]. Compared with PD, CRRT has a better capability of solute filtration and liquid removal efficiency, and PD cannot provide adequate clearance of toxic metabolites, which can be achieved with CRRT, especially in congenital metabolic diseases [25]. CRRT is particularly effective in maple syrup disease, organic acidemia with hyperammonemia, and inborn metabolism in urea cycle disorder [25–28]. Higher peak ammonia levels during hyperammonemia episodes are associated with worse survival rates, and the duration of coma before dialysis is negatively associated with cognitive outcomes [26, 29]. The rate of ammonia clearance with CRRT has been associated with improved outcomes [25–30]. Therefore, timely and aggressive treatment, including the use of dialysis, should be applied to rapidly lower ammonia levels [26–30]. In our study, CRRT was performed in 36 patients (25.5%) for metabolic reasons.

The frequency of CRRT performed mortality in children under 10 kg was 62% (total of 85 cases) in the study of Symons et al. [10], 38% (n = 16) in the study of Pedersen et al. [31], and 42.3% (71) in the study of Kaempfen et al. [13]. The mortality rate in our study was 48% (n = 141). Our study’s mildly higher mortality rate may be due to the high MODS rate (44%). The mortality rate of the patients with MODS in our study was 66.2%. MODS itself occurs in 30% to 50% of children in the PICU and is responsible for a disproportionately higher percentage of total deaths in PICUs, reaching over 90% in some studies [32]. In our study, patients who were treated with CRRT and died had higher PRISM scores, consistent with previous data [33,34].

One of the important indications of CRRT is fluid overload [1,31,35]. As an independent indicator of mortality, early initiation of CRRT has been recommended in patients with a >10% FO [36,37]. It has been reported that mortality increases as the percentage of FO increases when it passes 10% [31–39].

In our study, CVVH, CVVHD, and CVVHDF modes were used in CRRT, and these modes showed no significant differences between survivors and nonsurvivors. These data were similar to the findings of Symons et al. [10]. In the studies of Symons et al. [10] and Kaempfen et al. [13], CVVH mode was used in all patients. In our study, CVVHD mode was used more frequently in patients with a body weight of 5–10 kg.

The most common complications of CRRT in our study were hypotension (44.7%), hemorrhage (12.1%), electrolyte imbalance (58%), and hypothermia (58.2%), consistent with previous data [40–42].

The major limitation of this study was its retrospective design. We were not able to determine the exact reason for starting CRRT because we based it on what was documented in the patient records. In addition, the reason and timing of starting CRRT treatment may vary, especially according to the preference of the ‘pediatric intensivist’ in different centers. Also, we could not determine mechanical ventilation settings and ECMO flow rates. We could not detect circuit changes and circuit times from retrospective data; therefore, we could not compare circuit times between subgroups. No data on the exact cause of death are presented because some of the patients had multiple organ failures. The absence of a control group is another limitation.

## 5. Conclusion

To our knowledge, this multicenter study comprised the largest study group to date of children weighing less than or equal to 10 kg who underwent CRRT. Despite the technical difficulties of using modified equipment in critically ill children with a body weight of less than or equal to 10 kg, CRRT is a lifesaving extracorporeal treatment modality in dedicated and experienced PICUs. Although new CRRT devices are designed for younger children, access to these devices is restricted in most parts of the world. Especially in newborns, CRRT is an indispensable treatment method because it removes toxic substances faster than peritoneal dialysis in metabolic diseases.

## Figures and Tables

**Table 1:** Demographic variables and clinical course of CRRT: survivors vs. nonsurvivors.

Variable	Overall* (n = 141)	Survivors* (n = 73)	Nonsurvivors* (n = 68)	p-value
**Demographic variables**				
Male, n (%)	74 (52.5%)	37 (50.6%)	37 (54.4)	0.658
Weight (kg)	6 (4–8.35)	5 (3.5–7.7)	4 (3.16–5.95)	**0.010**
Age (month)	6 (2–12)	8 (4.25–14)	5 (2–11)	**0.026**
**Primary disease**				
Inborn error of metabolism disorder	36 (25.5)	24 (32.9)	12 (17.6)	0.038
Primary renal disease	21 (14.9)	18 (24.7)	3 (4.4)	0.001
Cardiac disease	18 (12.8)	4 (5.5)	14 (20.6)	0.007
Sepsis	35 (24.8)	15 (20.5)	20 (29.4)	0.223
Liver disease	4 (2.8)	0 (0)	4 (5.9)	0.052**
Pulmonary disease	21 (14.9)	7 (9.6)	14 (20.6)	0.067
Other diseases	6 (4.3)	5 (6.8)	1 (1.5)	0.210**
**PICU clinical data**				
Length of stay (day)	**10 (4**–**25)**	25.5 (18–33)	11.5 (7–23.5)	0.530
PRISM score	17 (10–27)	11 (8–21.5)	20 (14.75–34.25)	**<0.001**
PELOD score	22 (11–32)	12 (10–22.5)	31 (21–41.25	**<0.001**
The number of MODS	3 (1–4)	1 (1–3)	4 (3–4.75)	**<0.001**
Respiratory failure (%)	92 (65.2)	35 (47.9)	57 (83.8)	**0.001**
Cardiac failure (%)	80 (56.7)	24 (32.8)	56 (82.3)	**0.001**
Neurologic failure	38 (27)	15 (20.5)	23 (31.5)	0.076
Hematologic failure (%)	45 (31.9)	16 (21.9)	29 (39.7)	**0.010**
Renal failure (%)	83 (58.9)	37 (50.6)	46 (63)	**0.041**
Hepatic failure (%)	34 (24.1)	9 (12.3)	25 (36.7)	**0.001**
**CRRT indications (%)**				
Fluid overload	75 (53.2)	29 (39.7)	46 (73)	**0.001**
AKI	62 (44)	25 (34.2)	37 (54.4)	**0.016**
Oliguria	46 (32.6)	20 (27.3)	26 (38.2)	0.170
Hyperkalemia	9 (6.4)	4 (5.4)	5 (7.3)	0.738**
Acidosis	64 (45.4)	30 (41)	34 (50)	0.289
Metabolic disease	51 (36.2)	32 (43.8)	19 (27.9)	0.057
Hypo-hypernatremia	16 (11.3)	9 (12.3)	7 (10.2)	0.703
Electrolyte imbalance	49 (34.8)	20 (27.3)	29 (42.6)	0.057
Sepsis with MODS	62 (44)	21 (28.7)	41 (60.2)	**0.001**
Mechanical ventilation	115 (81.6)	47 (64.3)	68 (100)	**0.001**
ECMO	23 (16.3)	3 (4.1)	20 (29.4)	**0.001**
TPE	42 (29.7)	19 (26)	23 (33.8)	0.108

AKI: Acute kidney injury; CRRT: Continuous renal replacement therapy; Extracorporeal membrane oxygenation (ECMO); kg: Kilogram; MODS: Multi-organ dysfunction syndrome; PRISM III: Pediatric risk of mortality score, PELOD: Pediatric logistic organ dysfunction score; TPE: Therapeutic plasma exchange,

* median (IQR),

** Fisher’s exact test

**Table 2:** Demographic variables and clinical course of CRRT; survivors vs. nonsurvivors.

Variable	Total*(n = 141)	Survivors* (n = 73)	Nonsurvivors* (n = 68)	p-value
Days of CRRT	3 (1–5.07)	1.5 (0.85–3.35)	2.5 (1–5.25)	**0.004**
Number of circuits used	1.5 (1–3)	2 (1–3)	1 (1–3)	0.243
**CRRT catheter site (%)**				
RVJI	87 (61.7)	48 (65.8)	39 (57.4)	0.305
LVJI	20 (14.2)	13 (17.8)	7 (10.3)	0.201
Femoral	14 (9.9)	8 (11)	6 (8.8)	0.672
Subclavian	4 (2.8)	2 (2.7)	2 (2.9)	0.663**
ECMO	16 (11.3)	2 (2.7)	14 (20.5)	0.001
**Dialysis catheter size (%)**				
6.5 Fr	21 (16.8)	13 (17.8)	8 (11.7)	0.314
7 Fr	99 (79.2)	57 (78.1)	42 (61.8)	0.034
8 Fr	5 (4.0)	1 (1.3)	4 (5.9)	0.196
**CRRT modality (%)**				
CVVH	6 (4.3)	5 (6.8)	1 (1.4)	0.114
CVVHD	57 (40.4)	30 (41)	27 (39.7)	0.867
CVVHDF	78 (55.3)	38 (52)	40 (58.8)	0.419
**Type of CRRT circulation (%)**				
HF20	127 (90.1)	72 (98.6)	55 (80.8)	**0.001**
M60	14 (9.9)	1 (1.4)	13 (19.1)	**0.002** **
**Prime (%)**				
Packed red cell	96 (68.1)	41 (56.1)	55 (80.8)	**0.002**
Normal saline	40 (28.4)	31 (42.4)	9 (13.2)	<0.001
5% albumin	5 (3.5)	1 (1.4)	4 (5.8)	0.148
**Blood product during CRRT**				
Erythrocyte suspension	111 (78.7)	56 (76.7)	55 (80.8)	0.433
Thrombocyte suspension	73 (51.8)	26 (35.6)	47 (69.1)	**0.001**
Fresh frozen plasma	78 (55.3)	28 (38.3)	50 (73.5)	**0.001**
**CRRT complications**				
Hypotension	63 (44.7)	21 (28.7)	42 (61.7)	**0.001**
Hypothermia	51 (36.2)	21 (28.7)	30 (44.1)	**0.002**
Electrolyte imbalance	82 (58.2)	38 (52)	44 (64.7)	0.14
Infection	27 (19.1)	11 (15.1)	16 (23.5)	0.043
Transfusion	95 (67.4)	53 (72.6)	42 (61.7)	0.859
**Type of anticoagulant**				
Heparin	119 (84.4)	63 (86.3)	56 (82.3)	0.519
Citrate	5 (3.5)	2 (2.7)	3 (4.4)	0.592
No anticoagulant	17 (12.1)	8 (10.9)	9 (13.2)	0.678

CRRT: Continuous renal replacement therapy; CVVH: Continuous venovenous hemofiltration; CVVHD: Continuous venovenous hemodialysis; CVVHDF: Continuous venovenous hemodiafiltration, ECMO: Extracorporeal membrane oxygenation; LVJI: Left vena jugularis interna; RVJI: Right vena jugularis interna,

* median (IQR),

** Fisher’s exact test

**Table 3:** Demographic variables and Clinical course of CRRT; <5 kg vs. 5–10 kg.

Variable	Patients < 5 kg* (n = 52) (%)	Patients 5–10 kg* (n = 89) (%)	p
Male sex	28 (53.8)	46 (51.6)	0.804
Length of stay (IQR)	9.5 (4–32)	11 (4–25.5)	0.968
Days of CRRT (IQR)	3 (1.06–5.75)	3 (1–5.07)	0.754
Nonsurvivors	30 (57.6)	38 (42.6)	0.086
Acute kidney injury	21 (40.3)	41 (46)	0.512
Respiratory failure	42 (80.7)	50 (56.1)	**0.003**
Renal failure	25 (48)	58 (65.1)	0.047
Hepatic failure	13 (25)	21 (23.5)	0.851
Fluid overload	24 (46.1)	51 (57.3)	0.200
Oliguria	13 (25)	33 (37)	0.140
Metabolic disorder	27 (51.9)	44 (49.4)	0.003
Electrolyte imbalance	21 (40.3)	28 (31.4)	0.283
**Catheter site**			
RJVI	31 (59.6)	56 (62.9)	0.697
LJVI	5 (9.6)	15 (16.9)	0.235
Femoral	5 (9.6 )	9 (10.1)	0.924
Subclavian	3 (5.7)	1 (1.1)	0.142**
ECMO	8 (15.4)	8 (8.9)	0.248
**Dialysis catheter size**			
6.5 Fr	13 (25)	8 (8.9)	0.010
7 Fr	31 (59.6)	68 (76.4)	0.035
8 Fr	0 (0)	5 (5.6)	0.158
**Type of CRRT circuit**			
Hf20	44 (84.6)	82 (92.1)	0.056
M60	8 (15.3)	7 (7.8)	0.105
**CRRT modality**			
CVVH	3 (5.7)	3 (33.7)	0.496
CVVHD	15 (28.8)	42 (47.1)	**0.032**
CVVHDF	34 (65.3)	44 (49.4)	0.066
**Type of anticoagulant**			
Heparin	48 (92.3)	75 (84.2)	0.168
No anticoagulant	5 (9.6)	12 (13.4)	0.496
Citrate	0 (0)	5 (5.6)	0.082/0.158**
**Blood product during CRRT**			
Erythrocyte suspension	43 (82.6)	53 (59.5)	0.004
Thrombocyte suspension	26 (50)	47 (52.8)	0.625
Fresh Frozen plasma	31 (59.6)	47 (52.8)	0.403
**Complications**			
Hypotension	27 (51.9)	36 (43.8)	**0.028**
Thrombocytopenia	30 (57.6)	46 (51.6)	0.158
**Mechanical support**			
Mechanical ventilation	49 (94.2)	66 (74.1)	**0.003**
ECMO	12 (23)	11 (12.3)	0.085
TPE	16 (30.7)	26 (29.2)	0.848

CRRT: Continuous renal replacement therapy; CVVH: Continuous venovenous hemofiltration; CVVHD: Continuous venovenous hemodialysis; CVVHDF: Continuous venovenous hemodiafiltration, ECMO: Extracorporeal membrane oxygenation; LVJI: Left vena jugularis interna; RVJI: Right vena jugularis interna; TPE: Therapeutic plasma exchange,

* median (IQR),

** Fisher’s exact test

**Table 4:** Laboratory findings and vasoactive inotrope scores comparison of survivors and nonsurvivors groups before CRRT, on the first day of CRRT.

Variable	Before CRRT				First day of CRRT			
	n	S*	NS*	p	n	S*	NS*	p
BUN (mg/dL)	141	28.1 (11.45–49.5)	32.5 (15.4–47.7)	0.645	136	11 (3.75–20)	18 (7–24)	0.327
Creatinine (mg/dL)	140	0.94 (0.38–1.81)	0.86 (0.55–1.30)	0.608	137	0.56 (0.41–0.78)	0.66 (0.43–1.03)	0.843
Glucose (mg/dL)	128	102 (85–145.5)	134.5 (99.7–182.5)	**0.010**	127	122 (89–147.5)	131 (89–190)	0.131
Sodium (mmol/L)	141	140 (134–146)	143 (136–148.2)	0.146	138	140.5 (138.5–142.5)	142 (133–147)	0.177
Potassium (mmol/L)	141	3.8 (3.3–4.47)	3.7 (3.2–4.72)	0.733	136	3.37 (3.18–4.16)	3.33 (3.12–4.5)	**0.001**
Uric Acid (mg/dL)	127	9.2 (4.65–14.9)	6.1 (3–8.4)	**0.026**	117	3.55 (1.02–5.60)	3.8 (2.9–8.2)	0.768
Phosphor (mg/dL)	135	6.4 (5–8.7)	4.8 (3.9–7.2)	0.080	128	3.5 (2.3–4.5)	3.0 (2.38–4.67)	0.605
WBC (10^3^/μL)	141	12,200 (5950–17,875)	9700 (4210–16,500)	0.339	137	8700 (5280–13,800)	10,270 (4860–172,60)	0.575
Hemoglobin	141	9.5 (8.45–10.45)	10.3 (8.8–11.3)	0.311	137	9.5 (7.8–10.9)	10.2 (8.6–11.5)	**0.012**
Platelet (10^3^/μL)	141	182,000 (69,000–332,500)	71,000 (49,000–130,000)	**<0.001**	137	123.000 (48,000–201,000)	65,000 (45,250–120,500)	**<0.001**
pH	133	7.31 (7.18–7.39)	7.29 (7.19–7.37)	0.168	118	7.38 (7.34–7.42)	7.27 (7.22–7.36)	**<0.001**
HCO3	132	18.2 (11–23)	19.6 (15.4–24.9)	0.098	118	23 (21–26)	19.25 (14.80–23.32)	**<0.001**
Lactate (mmol/L)	130	3.7 (1.6–15.8)	8.8 (3.3–28.0)	**<0.001**	118	2.7 (1.2–6.6)	13.6 (6.17–46.75)	**<0.001**
Ammonia μ/dL	45	677.5 (241.25–1487)	521 (250–800)	0.623	29	122 (44.5–256)	198 (124–390)	0.791
VIS	42	6.5 (0–25.75)	55 (29.75–87)	**<0.001**	109	15 (10–20)	23 (13.13–39.25)	**<0.001**
Leucine (μmol/L) pre CRRT	9	1032 (152–1305)*						
Leucine (μmol/L) post CRRT					7	334 (103–468)*		

BUN: Blood urea nitrogen; VIS: Vasoactive inotrop score; WBC: White blood cell; S: Survivors; NS: Nonsurvivors;

* median (IQR)

**Table 5:** Comparison of laboratory findings and vasoactive inotrope scores of patients before CRRT and on the first day of CRRT.

Variable	n	Before CRRT*	n	First day of CRRT*	p
BUN (mg/dL)	141	27(10.8–46.8)	136	15.7(6.0–26.0)	**<0.001**
Creatinine (mg/dL)	140	0.88(0.49–1.55)	137	0.66(0.39–0.89)	**<0.001**
Glucose (mg/dL)	128	114.5(87.0–152.7)	127	118(98–146)	0.509
Sodium (mmol/L)	141	141(134.5–146)	138	140(136.7–143.3)	0.616
Potassium (mmol/L)	141	3.8(3.3–4.5)	136	3.3(3.0–4.1)	**<0.001**
Uric Acid (mg/dL)	127	6.5(4.1–11.0)	117	3.1(1.7–5.1)	**<0.001**
Phosphor (mg/dL)	135	5.1(4.2–6.9)	128	3.3(2.3–4.5)	**<0.001**
WBC (10^3^/μL)	141	11,000(5500–17,875)	137	8700(5200–14,875)	**0.009**
Hemoglobin	141	9.9(8.5–11.1)	137	10.3(8.7–11.7)	0.134
Platelet (10^3^/μL)	141	130,000(60,500–235,000)	137	76,000(43,000–131,500)	**<0.001**
pH	133	7.27(7.17–7.38)	118	7.34(7.24–7.40)	**<0.001**
HCO3	132	19(13.2–23.2)	118	22.0(18.5–25.2)	**<0.001**
Lactate (mmol/L)	130	5.2(2.2–16.0)	118	6.3(1.8–15)	0.291
Ammonia μ/dL	45	196(89.5–686)	29	124(45–247.5)	**<0.001**
VIS	42	22.5(0–51.3)	109	24.0(0–47.5)	**0.033**

BUN: Blood urea nitrogen; VIS: Vasoactive inotrop score; WBC: White blood cell;

* median (IQR)
